# Impact of digital health interventions on quality of life and mental health in older adults with chronic diseases: a systematic review and meta-analysis

**DOI:** 10.3389/fragi.2025.1737277

**Published:** 2026-01-05

**Authors:** Enliang Hu, Haozhe Wang, Jiayi Yao, Mingyu Liao, Wenjia Chen

**Affiliations:** 1 Department of Physical Training, Institute of Aviation Safety and Security, China Civil Aviation Flight Academy, Chengdu, China; 2 School of Physical Education, China University of Mining and Technology, Xuzhou, China

**Keywords:** chronic disease, digital health intervention, mental health, meta-analysis, older adults, quality of life, randomized controlled trial

## Abstract

**Background:**

Chronic diseases significantly impact the health of older adults globally. While digital health technologies offer new avenues for management, existing evidence remains inconsistent with methodological limitations.

**Methods:**

We systematically searched eight databases, including PubMed and Web of Science (from inception to 31 July 2025), for randomized controlled trials (RCTs) involving patients aged 60 and above with chronic conditions. A random-effects model was used to synthesize data to evaluate the impact of digital health interventions on general quality of life (QoL), disease-specific QoL, and mental health. The quality of evidence was assessed using the GRADE system.

**Results:**

Fifteen RCTs (n = 3253) were included. The meta-analysis showed that digital health interventions significantly improved general QoL (SMD = 0.54, 95% CI [0.30, 0.78]), disease-specific QoL (SMD = 0.39, 95% CI [0.17, 0.60]), and mental health status (SMD = 0.36, 95% CI [0.22, 0.50]) in older adults with chronic diseases. Although heterogeneity was observed in some outcomes, sensitivity analyses confirmed the robustness of the results. The GRADE assessment indicated that the quality of evidence for mental health improvement was high.

**Conclusion:**

Digital health interventions effectively enhance the quality of life and mental health of older adults with chronic diseases, demonstrating clear clinical significance. The evidence supports the integration of digital health technologies into routine care systems, particularly standardized interventions for patients with respiratory and metabolic diseases.

**Systematic Review Registration:**

https://www.crd.york.ac.uk/prospero/display_record.php?ID=CRD420251107861, identifier CRD420251107861.

## 1 Introduction

Chronic non-communicable diseases have become the most severe challenge facing global health (Piovani et al., 2022). This issue is particularly prominent among the older population because, with advancing age, physiological functions gradually decline, immunity decreases, and multimorbidity becomes common (Fang et al., 2020), while the ability to adapt to and recover from diseases significantly weakens (Lee and Choi, 2022), severely impairing multiple dimensions of their quality of life (Rizzuto et al., 2017). Quality of life, as a key indicator for measuring the effectiveness of medical interventions, is directly related to the happiness and life satisfaction of older adults, thereby influencing changes in physical function, activities of daily living, and social participation (Xu et al., 2020). At the same time, the negative impact of chronic diseases on mental health cannot be ignored; long-term disease burden easily triggers psychological issues such as depression and anxiety, further affecting the patient’s overall health status (Verhaak et al., 2005). According to United Nations projections, by 2050, older adults aged 65 and over will account for 16% of the global population (Fang et al., 2019). With the acceleration of global population aging, effectively improving the quality of life and mental health status of older adults with chronic diseases has become an urgent public health priority.

Digital health technologies provide an innovative path to address this challenge (Kitsiou et al., 2017). Currently, global mobile users have reached 5.8 billion, and the World Health Organization, in its Global Strategy on Digital Health 2020–2025, has explicitly positioned it as a key driver for achieving universal health coverage (World Health Organization, 2024; World Health Organization, 2023). DHIs are defined as interventions that use information and communication technologies, such as mobile applications, wearable devices, telemedicine platforms, virtual reality (VR), or exergames to support or directly provide health-related services (Marcolino et al., 2018; Hagelsrum et al., 2025). Although the application of digital technologies in chronic disease management is increasing (Abe et al., 2024; Kaddoussi et al., 2024), existing evidence presents a complex and inconsistent picture when focusing on quality of life as a core outcome. Previous systematic reviews have shown significant differences in intervention effects for patients with cancer or cardiovascular diseases (Zhuang et al., 2025), with some studies even reporting ineffective results (Buneviciene et al., 2021; Mohajeri et al., 2024). This inconsistency suggests that the impact of digital interventions on quality of life may be constrained by multiple factors such as intervention type, disease characteristics, and measurement tools (Brands et al., 2022; Yadav et al., 2019), necessitating high-quality evidence-based medicine for clarification.

A review of meta-analyses published in recent years reveals that, despite the increasing application of DHIs in chronic disease management for older adults, significant research gaps remain in the existing evidence. First, the handling of heterogeneity is insufficient. Previous studies often reported extremely high statistical heterogeneity (Bateman et al., 2016) but lacked effective sensitivity analysis strategies to identify the sources of outliers. Second, the distinction between dimensions is blurred. Most reviews conflated “general quality of life” with “disease-specific quality of life,” ignoring the differences in sensitivity to intervention response among more than 30 different measurement tools used, including SF-36 and EQ-5D, which compromised the comparability of results (Lopez-Alcalde et al., 2024; Cruz-Cobo et al., 2022). Finally, there is a lack of a cross-disease comparative perspective. Existing studies are mostly limited to single diseases, such as focusing solely on diabetes or cardiovascular disease, failing to answer which category of chronic disease patients benefits the most. Based on this, this study aims to evaluate the comprehensive impact of digital health interventions on the quality of life and mental health of older adults with chronic diseases through a systematic review and meta-analysis. The unique contributions of this study lie in: first, explicitly distinguishing and separately evaluating general versus disease-specific quality of life; second, resolving heterogeneity issues by eliminating statistical outliers through rigorous sensitivity analysis; and finally, applying the GRADE system to grade the quality of evidence across a cross-disease population for the first time. The results of this study will provide high-quality evidence for the formulation of personalized clinical digital prescriptions and the optimization of health policies.

## 2 Materials and methods

The protocol for this study has been registered with PROSPERO (Registration number: CRD420251107861), specifying the primary objectives, inclusion and exclusion criteria, intervention measures, control measures, and the primary and secondary outcome indicators planned for evaluation. The implementation of this systematic review strictly followed the pre-registered protocol without significant deviation and was conducted and reported in strict accordance with the Preferred Reporting Items for Systematic Reviews and Meta-Analyses (PRISMA 2020) checklist (Page et al., 2021).

### 2.1 Literature search and study selection

We systematically searched eight databases: PubMed, Web of Science, Scopus, Embase, Cochrane Library, CINAHL, MEDLINE, and CNKI. The search range covered the period from the inception of each database to 31 July 2025. Search terms were based on PICOS (Population, Intervention, Comparison, Outcome) and divided into Population (“older adults”, “chronic disease”, etc.), Intervention (“digital health”, “mHealth”, etc.), Comparison (“usual care”, “control group”, etc.), and Outcome (“quality of life”, “mental health”, etc.). The literature search strategy for the PubMed database is shown as an example in Table 1.

**TABLE 1:** Literature search strategy for PubMed database.

No.	Search strategy	Search field
1	Digital Health OR Telemedicine OR mHealth OR m-health OR mobile health OR eHealth OR e-health OR digital therapeutics OR digital intervention OR digital medicine OR remote monitoring OR telehealth OR digital platform OR mobile application OR mobile app OR smartphone app OR web based intervention OR internet based intervention OR online intervention OR computerized intervention OR digital technology OR wearable device OR health information technology OR virtual care	Title, Abstract
2	Aged OR elderly OR older adult OR older people OR senior OR geriatric OR advanced age OR 60 years OR 65 years OR over 60 OR over 65	Title, Abstract
3	Diabetes mellitus, type 2 OR type 2 diabetes OR diabetes mellitus OR non-insulin dependent diabetes OR NIDDM OR T2DM OR Hypertension OR Hypertension OR high blood pressure OR elevated blood pressure OR Cardiovascular Diseases OR cardiovascular disease OR heart disease OR coronary artery disease OR coronary heart disease OR cardiac disease OR CVD OR CHD OR pulmonary disease, chronic obstructive OR chronic obstructive pulmonary disease OR COPD OR chronic obstructive lung disease OR chronic airway obstruction	Title, Abstract
4	Mental Health OR Mental Health OR psychological health OR psychological wellbeing OR psychological wellbeing OR Depression OR Depression OR depressive OR Anxiety OR Anxiety OR psychological distress OR emotional health OR mood OR Quality of Life OR Quality of Life OR QoL OR life quality OR health-related quality of life OR HRQoL OR functional status OR wellbeing OR wellbeing OR life satisfaction	Title, Abstract
5	Randomized controlled trial OR controlled clinical trial OR randomized OR randomly OR trial OR control group OR intervention study OR clinical trial OR RCT	Title, Abstract
6	#1 AND #2 AND #3 AND #4 AND #5	​

### 2.2 Inclusion and exclusion criteria

Inclusion and exclusion criteria were determined based on the PICOS framework. Inclusion criteria were: (1) Participants were older adults aged 60 years and above; (2) The intervention group received various types of digital health interventions (wearable devices combined with other smart applications, telerehabilitation, digital health technologies, etc.); (3) The control group received routine care; (4) Outcome indicators included at least one quality of life or mental health indicator; (5) The study type was a randomized controlled trial (RCT). No restrictions on publication language were applied during the search process. Exclusion criteria were: (1) Animal experiments; (2) Non-randomized controlled trials (quasi-experiments, case-control trials, cross-sectional studies, etc.); (3) Qualitative studies, systematic reviews, meta-analyses, study protocols, gray literature, and abstracts; (4) Presence of uncontrolled activities outside the prescribed intervention; (5) Studies with incomplete data reporting or data that could not be extracted.

### 2.3 Study selection and data extraction

A customized data extraction tool was designed in Excel (Microsoft Inc., Redmond, WA, United States) to extract data. To ensure the accuracy of duplicate removal, the study primarily used EndNote software for literature management and deduplication. Duplicates were first identified using the automatic deduplication function of EndNote X9, followed by manual verification by researchers to ensure all duplicate records were removed. Two researchers independently screened the literature, first by reading titles and abstracts for preliminary screening, and then by reading the full text of potentially relevant studies to determine final inclusion. Disagreements were resolved through discussion or consultation with a third researcher if necessary. Data extraction used a pre-designed standardized form, including: (1) Study characteristics: first author, year of publication, country of study, study design, sample size; (2) Population characteristics: age, gender, type of chronic disease; (3) Intervention characteristics: intervention measures, intervention duration, follow-up time; (4) Outcome indicators: quality of life assessment tools, mental health assessment tools, baseline and endpoint values with their standard deviations, and number of events for dichotomous outcomes; (5) Other relevant information.

Data extraction and processing for analysis: For continuous variables, the mean and standard deviation (SD) of baseline and endpoint values for each group were prioritized, and the intervention effect was obtained by calculating the difference. When studies only reported 95% confidence intervals (CI), they were converted to standard deviations using the formula recommended by the Cochrane Handbook. For studies with loss to follow-up, the intention-to-treat (ITT) analysis principle was adopted, calculating effect sizes based on the original randomized sample size. When studies did not provide complete statistical parameters and these could not be obtained through standard conversion methods, the authors of the original studies were contacted via email to obtain raw data. If the authors did not respond or data could not be obtained, the study was excluded from the corresponding meta-analysis to ensure the accuracy and reliability of the analysis results. All data conversion processes were completed independently by two researchers and cross-verified, with disagreements resolved through discussion.

### 2.4 Quality assessment

The Risk of Bias 2 tool (RoB 2) recommended by the Cochrane Collaboration was used to evaluate the quality of included studies (Sterne et al., 2019), assessing five domains: bias arising from the randomization process, bias due to deviations from intended interventions, bias due to missing outcome data, bias in measurement of the outcome, and bias in selection of the reported result. Each domain was judged as “Low risk,” “Some concerns,” or “High risk,” and an overall risk of bias judgment was made for each study. The GRADE system was used to evaluate the quality of evidence (Guyatt et al., 2011; University, 2020), assessing downgrading factors across five aspects: risk of bias in study design, inconsistency, indirectness, imprecision, and publication bias. The quality of evidence was graded as High, Moderate, Low, or Very Low. Two reviewers independently completed the quality assessment, reaching consensus through discussion when opinions differed, or consulting third-party expert opinions if necessary.

### 2.5 Statistical analysis

Meta-analysis was performed using R software (version 4.5.0) with the metafor, forestplot, ggplot2, dplyr, and gridExtra packages. For continuous variables, the Standardized Mean Difference (SMD) was used as the effect size metric, using Hedges’ g correction to reduce small sample bias, and 95% confidence intervals were calculated. A random-effects model using Restricted Maximum Likelihood (REML) estimation was employed for the pooled analysis (Higgins et al., 2003). Heterogeneity between studies was assessed using the I^2^ statistic, Cochran’s Q test, and τ^2^ value, where I^2^ < 25%, 25%–50%, 50%–75%, and ≥75% indicated no/minor, low, moderate, and high heterogeneity, respectively. Sensitivity analysis used the “leave-one-out” method to evaluate result stability. Publication bias was comprehensively assessed using Egger’s linear regression test (P < 0.05 indicates potential bias), funnel plot visual assessment, and the trim-and-fill method. As the number of included studies for some outcome indicators was limited (<10), publication bias assessment results should be interpreted with caution. Subgroup analyses were conducted based on disease type, digital intervention type, and intervention duration (Laranjo et al., 2021; Yao et al., 2025). A random-effects model was used for each subgroup, and the moderating effect was assessed via the Q-test between subgroups. The forestplot package was used to draw forest plots, and the ggplot2 package was used to draw funnel plots and sensitivity analysis plots. All figures had a resolution of 300 DPI. A two-sided P < 0.05 was considered statistically significant. To ensure consistency in interpretation, for scales where lower scores indicate better health status, data were reversed prior to analysis. Therefore, in this study, a positive SMD (>0) consistently indicates a result favoring the intervention group.

## 3 Results

### 3.1 Literature search

**FIGURE 1:**
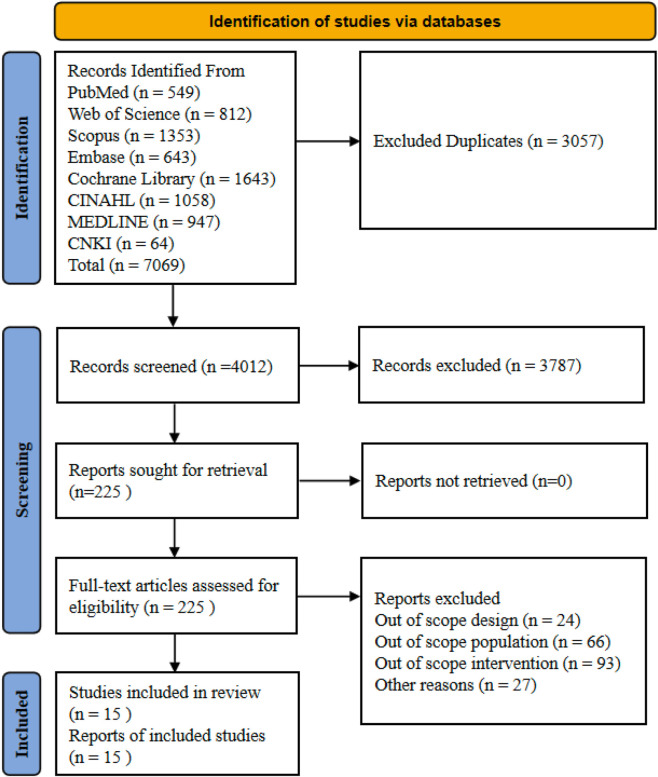
A flowchart of the literature search and study selection according to the PRISMA standard.

A total of 7,069 relevant articles were obtained through a systematic search of eight databases: PubMed, Web of Science, Scopus, Embase, Cochrane Library, CINAHL, MEDLINE, and CNKI. After automatically removing 3,057 duplicate articles using the automated reference management tool (EndNote X9), followed by manual verification by researchers to ensure accuracy, 4,012 articles were actually included for screening. After two researchers independently evaluated titles and abstracts, 3,787 articles not meeting the inclusion criteria were excluded, leaving 225 articles for the full-text assessment stage. Notably, full texts were successfully obtained for all 225 articles. During the full-text eligibility assessment, a further 210 articles were excluded. Reasons for exclusion mainly included: study design not meeting inclusion criteria (24), study population not meeting inclusion criteria (66), intervention measures not meeting inclusion criteria (93), and other reasons (27). Finally, this systematic review included 15 studies that met all inclusion criteria, all of which were included in the meta-analysis. The entire literature screening process strictly followed PRISMA statement requirements, ensuring the systematicity and transparency of the study, as shown in Figure 1.

### 3.2 Characteristics of included studies

This systematic review finally included 15 randomized controlled trials (see Table 2) (Bernocchi et al., 2018; Kempf et al., 2017; Tupper et al., 2018; Wong et al., 2022; Vorrink et al., 2016; Broers et al., 2020; Benzo et al., 2022; Victoria-Castro et al., 2024; Zamanillo-Campos et al., 2025; Blomqvist et al., 2025; Guillaumier et al., 2022; Jiang et al., 2025; Nishio et al., 2025; Bohingamu et al., 2019; Zanaboni et al., 2023), all of which were included in the meta-analysis. Publication dates ranged from 2016 to 2025 (Vorrink et al., 2016; Zamanillo-Campos et al., 2025). All studies employed a randomized controlled trial design, including international multi-center trials (Broers et al., 2020), multi-center trials (Benzo et al., 2022), and three-arm trials (Zamanillo-Campos et al., 2025). Sample sizes ranged from 20 to 674 cases (Victoria-Castro et al., 2024; Blomqvist et al., 2025), totaling 3,253 participants, with ages mainly concentrated between 60 and 77 years (Kempf et al., 2017; Wong et al., 2022). The included studies covered a variety of common chronic diseases, including 5 studies on chronic obstructive pulmonary disease (COPD) (Tupper et al., 2018; Vorrink et al., 2016; Benzo et al., 2022; Jiang et al., 2025; Zanaboni et al., 2023), 2 studies on heart failure (Victoria-Castro et al., 2024; Blomqvist et al., 2025), 2 studies on type 2 diabetes (Kempf et al., 2017; Zamanillo-Campos et al., 2025), 2 studies on multimorbidity (Wong et al., 2022; Bohingamu et al., 2019), and 1 study each for stroke (Guillaumier et al., 2022), coronary artery disease (Nishio et al., 2025), cardiovascular disease (Broers et al., 2020), and COPD combined with chronic heart failure (Bernocchi et al., 2018). Digital health interventions showed diverse characteristics, including online health behavior change platforms, mHealth applications, wearable device monitoring, remote monitoring systems, gamified rehabilitation interventions, and multi-component comprehensive interventions. Intervention duration ranged from 12 weeks to 12 months (Nishio et al., 2025; Zanaboni et al., 2023), with 12-week interventions being the most common (9 studies) (Bernocchi et al., 2018; Kempf et al., 2017; Wong et al., 2022; Broers et al., 2020; Benzo et al., 2022; Blomqvist et al., 2025; Guillaumier et al., 2022; Jiang et al., 2025; Nishio et al., 2025). Most studies set a follow-up period of 12–24 weeks (Guillaumier et al., 2022; Jiang et al., 2025). Control groups mainly received routine care or standard care, while others used daily activities or waiting list routine care. All studies used health-related quality of life as a primary or secondary outcome indicator, utilizing measurement tools including general scales (SF-36, SF-12, EQ-5D series, AQoL-8D, 15D questionnaire, etc.) and disease-specific scales (KCCQ, CRQ, CAT, MLHFQ, etc.).

**TABLE 2:** A list of basic characteristics of the studies included in the meta-analysis.

Author (Year)	Study design	Sample size	Age	Intervention	Intervention duration	Control group	Measurement tools	Disease type
Guillaumier et al. (2022)	Prospective randomized controlled trial	199/200	67 ± 12/68 ± 12	Online health behavior change intervention	12 weeks, 24-week follow-up	Usual health intervention group	EQ-VAS, EQ-5D-5L	Stroke
Jiang et al. (2025)	Three-arm parallel design randomized controlled trial	49/49/49	75.44 ± 4.48/75.73 ± 5.32/74.71 ± 4.71	Gamification-based pulmonary rehabilitation combined with Health Action Process Approach theory/Health Action Process Approach theory-based pulmonary rehabilitation	12-week intervention, 12-week follow-up	Usual pulmonary rehabilitation group	CAT	COPD
Blomqvist et al. (2025)	Parallel design pilot randomized controlled trial	10/10	78 ± 9/77 ± 5	Activity Coach app combined with wearable device	12 weeks	Usual care	KCCQ	HF
Bohingamu et al. (2019)	Pilot randomized controlled trial	83/80	70.70 ± 11.56/70.13 ± 13.26	Home-based personalized telemedicine intervention	24 weeks	Usual care	AQoL-8D	Diabetes and/or COPD
Nishio et al. (2025)	Randomized controlled trial	24/25	63.8 ± 6.4/62.6 ± 7.9	Home exercise program based on online guided cardiopulmonary exercise testing (CPET)	12 weeks	Usual health intervention group	SF-36	CAD
Zanaboni et al. (2023)	International multicenter randomized controlled trial	40/40/40	64.9 ± 7.1/64.0 ± 7.7/63.5 ± 8.0	Telerehabilitation group/Home training group	24 months	Usual care	EQ-5D, CAT, EQ-VAS	COPD
Zamanillo-Campos et al. (2025)	Two-arm randomized controlled trial	334/340	65 ± 10	DiabeText customized text message intervention	48 weeks	Usual care	EQ-5D-5L, DSES, MEDAS-14	Type 2 diabetes
Victoria-Castro et al. (2024)	Four-arm parallel group randomized controlled trial	46/46/46/44	Median age 61 years	Bodyport smart scale-based intervention/Conversa automated dialogue platform-based/Noom smartphone coach app-based	13 weeks	Usual care	KCCQ	Heart failure
Benzo et al. (2022)	Multicenter randomized controlled trial	188/187	69.3 ± 9.5/68.7 ± 9.5	Remote monitoring combined with health coaching intervention	12 weeks, 12-week follow-up	Usual care	CRQ	COPD
Vorrink et al. (2016)	Multicenter randomized controlled trial	84/73	62 ± 9/63 ± 8	Multi-platform integrated remote monitoring intervention	24 weeks	Usual care	CRQ-SAS	COPD
Broers et al. (2020)	International multicenter randomized controlled trial	74/75	63.26 ± 8.35/63.88 ± 8.30	Personalized e-health intervention	12 weeks	Usual care	WHOQOL-BREF	Cardiovascular disease
Wong et al. (2022)	Three-arm single-blind randomized controlled trial	74/71/76	74.7 ± 7.6/77.6 ± 7.8/77.4 ± 8.2	Mobile health app with nurse case management/Mobile health app	12 weeks	Daily activities	SF-12v2, GDS-15	Community-dwelling elderly with chronic pain, hypertension, or diabetes
Tupper et al. (2018)	Single-center randomized controlled trial	141/140	69.8 ± 9.0/69.4 ± 10.1	Multi-platform integrated remote monitoring intervention	24 weeks	Usual care	15D, CAT	COPD
Kempf et al. (2017)	Single-center randomized controlled trial	102/100	59 ± 9/60 ± 8	Telemedicine lifestyle intervention	12 weeks	Usual care	SF-12	Type 2 diabetes
Bernocchi et al. (2018)	Multicenter randomized controlled trial	56/56	71 ± 9/70 ± 9.5	Home telerehabilitation intervention	12 weeks	Usual care	MLHFQ, CAT	COPD, CHF

### 3.3 Quality assessment

**FIGURE 2:**
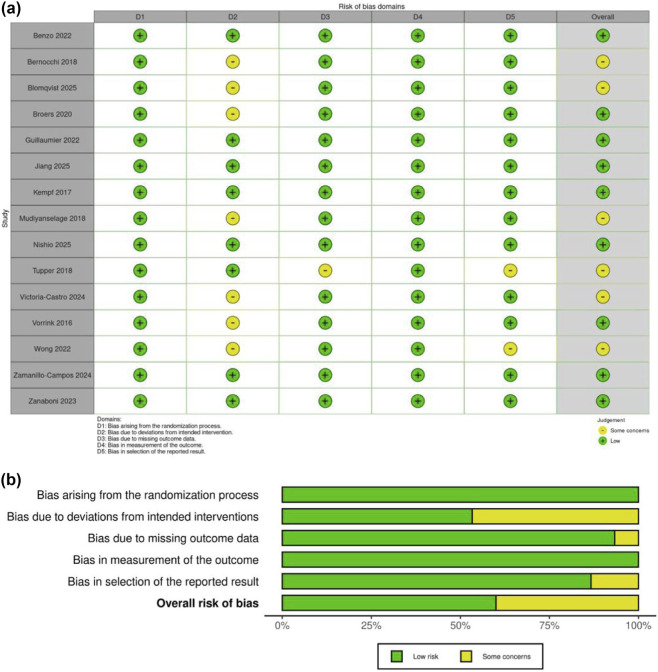
**(a)** Risk of bias summary: review of the authors judgments about each risk of bias item for each included study. **(b)** Risk of bias graph: review authors’ judgments about each risk of bias item, presented as percentage of included studies.

This study used the Cochrane Risk of Bias tool version 2.0 (RoB 2.0) to evaluate the methodological quality of the 15 included RCTs (Figure 2). Assessment results showed that all studies were at low risk in the domains of the randomization process (D1) and measurement of the outcome (D4), indicating that the generation of random sequences, allocation concealment, and outcome measurement employed standardized methods and were executed normatively. In the domain of deviations from intended interventions (D2), 8 studies (53.3%) were low risk, while 7 studies (46.7%) had some concerns, mainly stemming from the inherent difficulty of implementing blinding in digital health intervention studies. regarding missing outcome data (D3), 14 studies (93.3%) were low risk, with only 1 study having concerns due to improper handling of missing data. In the selection of the reported result domain (D5), 13 studies (86.7%) were low risk, while 2 studies had concerns due to a lack of pre-registered protocols or incomplete reporting of secondary outcomes. The overall risk of bias assessment showed that 9 studies (60%) were low risk, 6 studies (40%) had some concerns, and no studies were high risk. Overall, the methodological quality of the included studies was good, with concerns mainly concentrated on intervention blinding and the completeness of outcome reporting, which does not affect the overall reliability of the meta-analysis results.

## 4 Meta-analysis

### 4.1 Quality of life

#### 4.1.1 General quality of life

**FIGURE 3:**
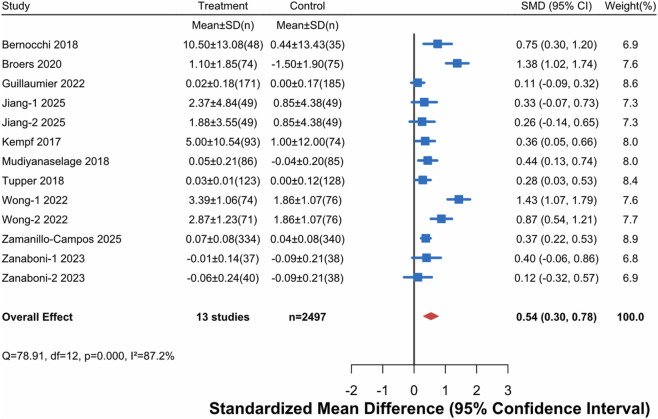
Forest Plot for Generic Quality of Life. Note: An effect size (SMD) > 0 indicates a result favoring the digital health intervention group.

This analysis included general quality of life data from 10 studies. Among them, the studies by Jiang (2025), Wong (2022), and Zanaboni (2023) were three-arm trials, each containing two independent intervention groups; thus, 13 comparisons were actually included. The studies involved a total of 1,249 patients in the intervention groups and 1,248 patients in the control groups. Meta-analysis using a random-effects model showed (Figure 3) that digital health interventions significantly improved general quality of life in older adults with chronic diseases (SMD = 0.54, 95% CI [0.30, 0.78]). According to Cohen’s criteria, this effect size is considered moderate and clinically significant. However, high heterogeneity existed between studies (I^2^ = 87.2%, df = 12, P = 0.000), suggesting considerable variation in study results.

#### 4.1.2 Disease-specific quality of life

**FIGURE 4:**
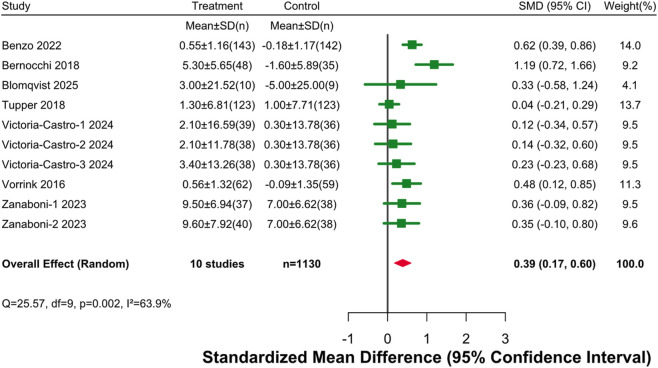
Forest Plot for Disease-Specific Quality of Life. Note: An effect size (SMD) > 0 indicates a result favoring the digital health intervention group.

This analysis included disease-specific quality of life data from 7 studies. Among them, Victoria-Castro (2024) was a four-arm trial containing three independent intervention groups; Zanaboni (2023) was a three-arm trial containing two independent intervention groups; thus, 10 comparisons were actually included. The studies involved a total of 578 patients in the intervention groups and 552 patients in the control groups. Meta-analysis using a random-effects model showed (Figure 4) that digital health interventions significantly improved disease-specific quality of life in older adults with chronic diseases (SMD = 0.39, 95% CI [0.17, 0.60]). According to Cohen’s criteria, this effect size belongs to the small-to-moderate range. Moderate to high heterogeneity existed between studies (I^2^ = 63.9%, df = 9, P = 0.002), suggesting certain differences in study results.

### 4.2 Mental health

**FIGURE 5:**
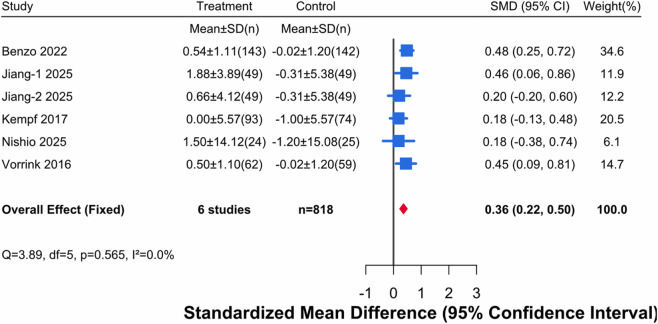
Forest Plot for Mental Health. Note: An effect size (SMD) > 0 indicates a result favoring the digital health intervention group.

This analysis included mental health score data from 5 studies, containing 6 comparisons, involving a total of 420 patients in the intervention groups and 398 patients in the control groups. The heterogeneity test showed extremely low heterogeneity (I^2^ = 1.3%, P = 0.565). Although statistical heterogeneity was not significant, given that the included studies were not functionally identical in terms of digital intervention forms and implementation details, a random-effects model was adopted for the pooled analysis to avoid underestimating the uncertainty of interval estimates. The results showed (Figure 5) that digital health interventions significantly improved the mental health status of older adults with chronic diseases (SMD = 0.36, 95% CI [0.22, 0.50]).

### 4.3 Sensitivity analysis

**FIGURE 6:**
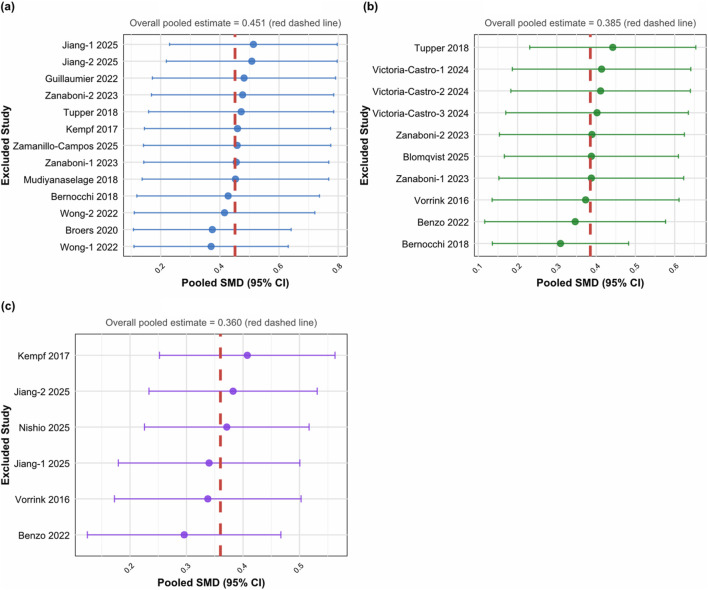
Sensitivity analysis. **(a)** General quality of life. **(b)** Disease-specific quality of life. **(c)** Mental health.

Sensitivity analysis was performed on primary outcome indicators using the “leave-one-out” method (Figure 6). In the general quality of life analysis, the pooled effect size ranged from 0.35 to 0.55 after sequentially removing each study, and all 95% confidence intervals did not cross the line of no effect. The analysis of disease-specific quality of life showed that the effect size remained within a reasonable range after removing individual studies, without extreme changes. In the mental health analysis, the variation in effect size after removing each study was controlled between 0.30 and 0.40, indicating good stability. Sensitivity analysis confirmed the robustness of the main results, with no outcome indicators undergoing substantive changes due to the removal of a single study.

### 4.4 Publication bias

**FIGURE 7:**
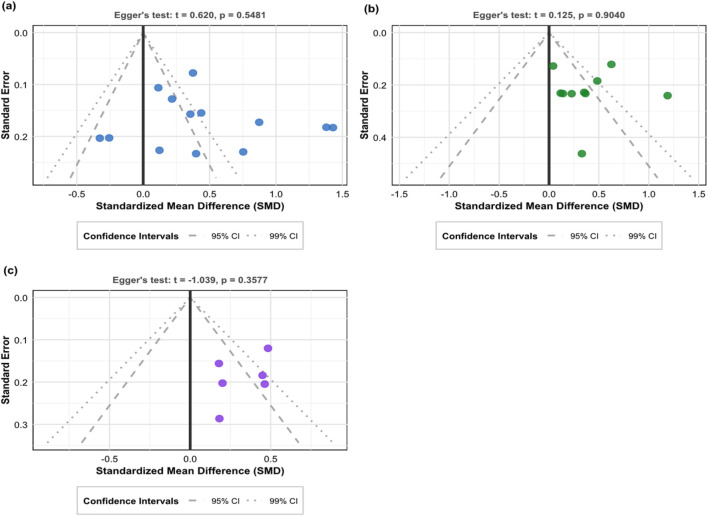
Funnel plot for Egger’s test. **(a)** General quality of life, **(b)** Disease-specific quality of life, **(c)** Mental health.

Egger’s test was used to assess potential publication bias (Figure 7). The results of Egger’s test showed that general quality of life (t = 0.620, P = 0.548), disease-specific quality of life (t = 0.125, P = 0.904), and mental health (t = −1.039, P = 0.358) did not reach statistical significance, suggesting no obvious publication bias. However, visual assessment of the funnel plot showed mild asymmetry, especially for the mental health outcome, where small-sample studies were unevenly distributed in the lower left of the funnel plot. Given the complexity of publication bias and the limitations of Egger’s test in small sample situations, the trim-and-fill method was further used for verification.

**FIGURE 8:**
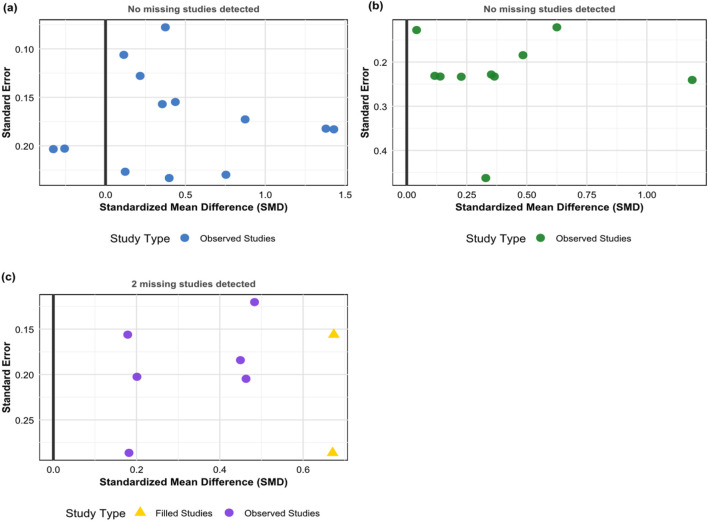
Trim-and-fill analysis. **(a)** General quality of life, **(b)** Disease-specific quality of life, **(c)** Mental health.

The trim-and-fill results showed (Figure 8) that no missing studies were detected for general quality of life and disease-specific quality of life, while 2 potentially missing small-sample negative result studies were detected for mental health. It must be acknowledged that the number of studies included in this research is relatively limited (5–13 studies for each outcome indicator), with some being below the recommended threshold of 10 studies for publication bias assessment. Although the power of statistical tests is limited, the comprehensive analysis combining visual assessment and the trim-and-fill method suggests that the impact of publication bias on the main conclusions of this study is relatively limited.

### 4.5 Subgroup analysis

To explore potential sources of heterogeneity between studies and evaluate the robustness of intervention effects under different conditions, this study conducted subgroup analyses on general quality of life and disease-specific quality of life based on three pre-defined dimensions: disease type, digital intervention type, and intervention duration (Table 3).

**TABLE 3:** Summary table of subgroup analysi**s**.

Subgroups	Outcomes	No. Of studies	SMD (95% CI)	P-value for overall effect	I^2^ for heterogeneity	P-value for heterogeneity
Disease categories						
Respiratory diseases	General quality of life	6	0.31 [0.16, 0.46]	P = 0.00	0.00%	P = 0.39
	Diseasespecific quality of life	5	0.36 [0.14, 0.59]	P = 0.00	57.01%	P = 0.04
Cardiovascular diseases	General quality of life	1	1.38 [1.02, 1.74]	P = 0.00	-	-
	Diseasespecific quality of life	5	0.40 [0.02,0.83]	P = 0.06	71.29%	P = 0.01
Metabolic diseases	General quality of life	3	0.38 [0.26, 0.51]	P = 0.00	0.00%	P = 0.92
Other chronic diseases	General quality of life	3	0.79 [0.04, 1.55]	P = 0.04	94.83%	P = 0.00
Digital intervention types						
Mobile applications/platform-based	General quality of life	4	0.68 [0.12, 1.25]	P = 0.02	95.43%	P = 0.00
	Diseasespecific quality of life	4	0.17 [−0.07, 0.42]	P = 0.17	0%	P = 0.97
Remote monitoring with guidance	General quality of life	2	0.79 [−0.35, 1.93]	P = 0.17	96.36%	P = 0.00
	Diseasespecific quality of life	3	0.36 [0.02, 0.70]	P = 0.04	78.09%	P = 0.01
Home-based rehabilitation programs	General quality of life	7	0.38 [0.23, 0.52]	P = 0.00	0.00%	P = 0.59
	Diseasespecific quality of life	3	0.63 [0.09, 1.17]	P = 0.02	76.29%	P = 0.02
Intervention duration						
Short-term intervention (12–13 weeks)	General quality of life	8	0.68 [0.32, 1.04]	P = 0.00	89.10%	P = 0.00
	Diseasespecific quality of life	6	0.44 [0.11, 0.78]	P = 0.01	71.30%	P = 0.01
Medium-term intervention (24 weeks)	General quality of life	2	0.31 [0.10, 0.52]	P = 0.00	17.55%	P = 0.27
	Diseasespecific quality of life	2	0.24 [−0.19, 0.68]	P = 0.27	74.61%	P = 0.05
Long-term intervention (≥48 weeks)	General quality of life	3	0.35 [0.22,0.49]	P = 0.00	0.00%	P = 0.56
	Diseasespecific quality of life	2	0.36 [0.04, 0.67]	P = 0.03	0.00%	P = 0.97

#### 4.5.1 Subgroup analysis based on disease type

Regarding general quality of life, the respiratory disease group (6 studies) showed a statistically significant improvement (SMD = 0.31, 95% CI [0.16, 0.46], P < 0.001), with no intragroup heterogeneity (I^2^ = 0.00%, P = 0.39). The metabolic disease group (3 studies) also showed consistent improvement effects (SMD = 0.38, 95% CI [0.26, 0.51], P < 0.001, I^2^ = 0.00%, P = 0.92). The cardiovascular disease group contained only 1 study, showing a large effect size (SMD = 1.38, 95% CI [1.02, 1.74], P < 0.001). The “other chronic diseases” group (3 studies), although showing a large effect size (SMD = 0.79, 95% CI [0.04, 1.55], P = 0.04), had extremely high heterogeneity (I^2^ = 94.83%, P < 0.001). Regarding disease-specific quality of life, the respiratory disease group (5 studies) showed significant improvement (SMD = 0.36, 95% CI [0.14, 0.59], P < 0.001), with moderate heterogeneity (I^2^ = 57.01%, P = 0.04). The cardiovascular disease group (5 studies) showed improvement at a borderline significant level (SMD = 0.40, 95% CI [0.02, 0.83], P = 0.06), with high heterogeneity (I^2^ = 71.29%, P = 0.01).

#### 4.5.2 Subgroup analysis based on digital intervention type

Regarding general quality of life, home-based rehabilitation program interventions (7 studies) showed robust improvement effects (SMD = 0.38, 95% CI [0.23, 0.52], P < 0.001), with no heterogeneity (I^2^ = 0.00%, P = 0.59). Mobile application/platform-based interventions (4 studies) showed moderate improvement (SMD = 0.68, 95% CI [0.12, 1.25], P = 0.02), but intragroup heterogeneity was extremely high (I^2^ = 95.43%, P < 0.001). Remote monitoring with guidance interventions (2 studies) had the largest numerical effect size but did not reach statistical significance (SMD = 0.79, 95% CI [-0.35, 1.93], P = 0.17), and heterogeneity was significant (I^2^ = 96.36%, P < 0.001). Regarding disease-specific quality of life, home-based rehabilitation program interventions (3 studies) showed a large improvement effect (SMD = 0.63, 95% CI [0.09, 1.17], P = 0.02), with high heterogeneity (I^2^ = 76.29%, P = 0.02). Remote monitoring with guidance interventions (3 studies) showed small-to-moderate improvement (SMD = 0.36, 95% CI [0.02, 0.70], P = 0.04, I^2^ = 78.09%, P = 0.01). Mobile application/platform-based interventions (4 studies) did not show statistically significant effects (SMD = 0.17, 95% CI [-0.07, 0.42], P = 0.17, I^2^ = 0.00%, P = 0.97).

#### 4.5.3 Subgroup analysis based on intervention duration

Regarding general quality of life, the short-term intervention group (12–13 weeks, 8 studies) had the largest effect size (SMD = 0.68, 95% CI [0.32, 1.04], P < 0.001), accompanied by high heterogeneity (I^2^ = 89.10%, P < 0.001). The medium-term intervention group (24 weeks, 2 studies) and long-term intervention group (≥48 weeks, 3 studies) had relatively smaller effect sizes (SMD = 0.31 and 0.35, respectively), but lower heterogeneity (I^2^ = 17.55% and 0.00%, respectively). Regarding disease-specific quality of life, the short-term intervention group (6 studies) showed moderate improvement (SMD = 0.44, 95% CI [0.11, 0.78], P = 0.01, I^2^ = 71.30%). The long-term intervention group (2 studies) showed significant improvement with no heterogeneity (SMD = 0.36, 95% CI [0.04, 0.67], P = 0.03, I^2^ = 0.00%). The medium-term intervention group (2 studies) did not reach statistical significance (P = 0.27).

### 4.6 Summary of evidence quality

This study used the GRADE (Grading of Recommendations Assessment, Development and Evaluation) system to comprehensively assess the evidence quality of each outcome indicator. GRADE assessment considered downgrading factors such as risk of bias, inconsistency, indirectness, imprecision, and publication bias, as well as upgrading factors such as effect size magnitude, dose-response relationship, and confounding factors, as shown in Table 4.

**TABLE 4:** Evidence quality assessment based on GRADE system.

Outcome indicator	Number of studies	Effect size [95% CI]	Risk of bias	Inconsistency	Indirectness	Imprecision	Publication bias	Evidence quality level
	(Participants)							
Generic quality of life	13 comparisons (2497 cases)	SMD = 0.54 [0.30, 0.78]	Not downgraded (a)	Downgraded (b)	Not downgraded	Not downgraded	Not downgraded (c)	⊕⊕⊕⊖ Moderate
Disease-specific quality of life	10 comparisons (1130 cases)	SMD = 0.39 [0.17, 0.60]	Not downgraded (a)	Downgraded (d)	Not downgraded	Not downgraded	Not downgraded (c)	⊕⊕⊕⊖ Moderate
Mental health	6 comparisons (818 cases)	SMD = 0.36 [0.22, 0.50]	Not downgraded (a)	Not downgraded (e)	Not downgraded	Not downgraded	Cannot be assessed (f)	⊕⊕⊕⊕ High

Footnote explanations: a. 60% of studies at low risk of bias, 40% of studies raised some concerns, mainly due to inherent difficulties in implementing blinding in digital health intervention research, but did not affect result reliability; b. High heterogeneity present (I^2^ = 87.2%, P < 0.001), downgraded one level for inconsistency; c. Egger’s test did not reach statistical significance (generic quality of life P = 0.548; disease-specific quality of life P = 0.904), funnel plot basically symmetric; d. Moderate to high heterogeneity present (I^2^ = 63.9%, P = 0.002), downgraded one level for inconsistency; e. No heterogeneity (I^2^ = 0%, P = 0.565), highly consistent results; f. Number of included studies <10, cannot conduct reliable publication bias assessment.

## 5 Discussion

### 5.1 Summary of evidence

This systematic review and meta-analysis synthesized 15 high-quality randomized controlled trials to comprehensively evaluate the impact of digital health interventions on the quality of life and mental health of older adults with chronic diseases. The results indicate that digital health interventions produced statistically significant and clinically meaningful improvements across all three core outcomes. First, regarding general quality of life, the primary analysis revealed a moderate improvement (SMD = 0.54, 95% CI [0.30, 0.78]). This effect size exceeds the commonly accepted threshold for the Minimal Clinically Important Difference (MCID, 0.3–0.5), indicating that patients can perceive a tangible uplift in their health status. Second, regarding disease-specific quality of life, the intervention group showed significant improvement over the control group (SMD = 0.39, 95% CI [0.17, 0.60]); although this effect size was slightly lower than that of general quality of life, this may reflect that improving specific disease symptoms is more challenging than enhancing general subjective wellbeing. Finally, the mental health dimension demonstrated the most consistent improvement (SMD = 0.36, 95% CI [0.22, 0.50]). Notably, according to the GRADE assessment, the quality of evidence for the mental health outcome was rated as “High,” suggesting a high degree of confidence and stability in this finding.

### 5.2 Comparison with previous studies and interpretation

Research in recent years regarding the impact of digital health interventions on older adults with chronic diseases has presented a complex and inconsistent pattern of results. For example, a systematic review by Park et al. (2025) targeting older adults living alone with chronic diseases found that digital health interventions did not reach statistical significance for improving general quality of life or depression symptoms (Kanai et al., 2025); Kanai et al. (2025) also noted that mHealth had limited long-term improvement effects on quality of life (Park et al., 2025). The reasons for these inconsistent results or low effect sizes in previous studies may lie in the inclusion of literature with high heterogeneity without sufficient subgroup differentiation. In contrast, by including a broader population of older adults with chronic diseases and employing rigorous sensitivity analyses, this study found more consistent and significant effects, with effect sizes for general quality of life, disease-specific quality of life, and mental health all reaching moderate levels with robust evidence.

Through sensitivity analysis and case-by-case review, this study identified sources of heterogeneity. First is the study population and disease characteristics. Subgroup analysis showed low heterogeneity (I^2^ = 0%) in the respiratory and metabolic disease groups for general quality of life improvement. This may be attributed to the clear physiological monitoring indicators (e.g., blood glucose, blood oxygen) and standardized management pathways associated with these diseases, making patient responses to digital interventions relatively consistent. In contrast, the “multimorbidity” or “other chronic diseases” groups showed significant heterogeneity. Taking the excluded study by Wong (2022) as an example, it included a population with multimorbidity accompanied by chronic pain; such complex health needs led to differences in intervention response. Second is the intervention mode. This study found that although mobile application/platform-based interventions had larger effect sizes, they showed significant heterogeneity; this may be related to differences in user interface design and engagement across different apps. Finally, measurement tools. The heterogeneity in disease-specific quality of life (which dropped to 0% after excluding outliers) suggests that different scales, such as the KCCQ for heart failure versus the CAT for COPD, have different sensitivities to interventions. In summary, the heterogeneity observed in this study reflects the variation of digital health interventions across different disease backgrounds, population characteristics, and implementation modes. This finding explains why previous studies yielded weak effects due to a failure to exclude confounding factors, and also suggests that future clinical applications should customize intervention plans based on patients’ specific comorbidity status rather than adopting a one-size-fits-all strategy.

### 5.3 Implications for practice

The effects of digital health interventions found in this study have important clinical value. The significant improvement in general quality of life, classified as a moderate effect according to Cohen’s criteria and exceeding the MCID threshold of 0.3, indicates that patients can actually perceive an enhancement in quality of life (Norman et al., 2003). Disease-specific quality of life and mental health also achieved robust improvements, and the high consistency of mental health results is particularly rare in the generally high-heterogeneity field of digital health. These findings support the use of digital health technologies as an effective supplement to traditional care for improving the overall wellbeing of older adults with chronic diseases.

The exploratory results based on subgroup analysis provide some preliminary clues for clinical practice, but should be interpreted with caution. Data show that patients with respiratory and metabolic diseases demonstrate statistically high consistency in response to digital interventions, which may suggest that standardized digital interventions are easier to replicate and promote for diseases with clear physiological monitoring needs. Additionally, home-based rehabilitation program interventions showed a trend of lower heterogeneity in improving general quality of life, which might imply that structured processes help control implementation quality. It must be emphasized that the above analysis regarding intervention characteristics is observational in nature, and some subgroups (e.g., cardiovascular disease group, medium-term intervention group) included very few studies (<3), limiting statistical power. Therefore, these findings should be viewed as hypothesis-generating rather than definitive clinical conclusions. In actual decision-making, clinicians should not blindly rely on single subgroup results but should conduct individualized assessments combining the patient’s specific comorbidity status, technology acceptance, and accessibility of medical resources. For complex patients with multimorbidity, existing high-heterogeneity data suggest the need to explore more flexible or hybrid intervention models involving human support, which requires further verification in future large-sample studies.

### 5.4 Limitations and future perspectives

Despite adopting strict methodological standards, this study still has several key limitations that need to be considered when interpreting the results. First, there are limitations at the data and measurement level: this study excluded literature with incomplete data reporting where raw data could not be obtained, which may introduce potential selection bias; meanwhile, the included studies used a variety of heterogeneous quality of life measurement tools (e.g., SF-36, EQ-5D, and various specific scales). Although standardized mean differences were used for synthesis, intrinsic differences between scales still reduce the comparability of results. It is particularly important to point out that although high statistical consistency (I^2^ = 0%) was achieved after excluding outliers based on sensitivity analysis, there is a risk of artificially inflating result consistency. The excluded studies often reflect the intervention status of complex populations in the real world (such as those with multimorbidity), and completely excluding these data may limit the generalizability of the results. Furthermore, most included studies had short follow-up periods (12–24 weeks), and the difficulty of implementing double-blinding due to the characteristics of digital interventions limits judgments on long-term efficacy and the objectivity of results.

Addressing these limitations, future research should focus on improving the quality and depth of evidence. It is recommended that this field establish a standardized core outcome set and unify quality of life assessment frameworks to improve the efficiency of cross-study evidence synthesis. Simultaneously, future randomized controlled trials should extend follow-up times (at least 12 months) to evaluate the long-term maintenance effects of digital interventions on behavior change in older adults with chronic diseases. In addition, future research should strive to go beyond simple validity verification and delve into the internal mechanisms of intervention efficacy, using multi-arm trial designs to precisely analyze the specific contributions of technical components such as real-time feedback, remote guidance, or social support to improving quality of life, thereby providing a solid scientific basis for precision digital medicine.

## 6 Conclusion

This systematic review and meta-analysis, based on 15 randomized controlled trials, established the clinical value of digital health interventions in improving the quality of life and mental health of older adults with chronic diseases. Evidence indicates that these interventions significantly enhanced patients’ general quality of life (SMD = 0.54), disease-specific quality of life (SMD = 0.39), and mental health status (SMD = 0.36). Furthermore, after excluding sources of heterogeneity, the results demonstrated high consistency, providing GRADE high-quality evidence for the mental health benefits in this field. The digital technologies evaluated in this study are extensive, covering mobile applications, wearable monitoring devices, and telerehabilitation platforms. comprehensively, schemes with structured processes and sustained support demonstrated better application prospects and stability, particularly with respiratory and metabolic disease patients showing more consistent responses to standardized digital interventions. This finding suggests that future remote medical project designs should prioritize long-term intervention mechanisms and standardized management. Therefore, digital health interventions, especially those capable of providing precise guidance combined with disease characteristics, offer new possibilities for the personalized and remote management of chronic diseases in older adults. From a public health and policy perspective, the findings of this study have positive implications. Considering the inherent scalability and accessibility of digital interventions, it is recommended that policymakers integrate them into national chronic disease management strategies and medical insurance reimbursement systems, and formulate precise digital prescriptions based on disease characteristics, thereby providing a sustainable and affordable supplementary strategy to address the challenge of medical resource scarcity in the context of global aging.

## Data Availability

The original contributions presented in the study are included in the article/supplementary material, further inquiries can be directed to the corresponding author.
